# Improvement of soil acidification in tea plantations by long-term use of organic fertilizers and its effect on tea yield and quality

**DOI:** 10.3389/fpls.2022.1055900

**Published:** 2022-12-23

**Authors:** Jianghua Ye, Yuhua Wang, Yuchao Wang, Lei Hong, Xiaoli Jia, Jiaqian Kang, Shaoxiong Lin, Zeyan Wu, Haibin Wang

**Affiliations:** ^1^ College of Tea and Food, Wuyi University, Wuyishan, China; ^2^ College of Life Science, Fujian Agriculture and Forestry University, Fuzhou, China; ^3^ College of Life Science, Longyan University, Longyan, China

**Keywords:** tea plantation, organic fertilizer, soil acidification, yield, quality

## Abstract

Soil acidification in tea plantation seriously reduced the yield and quality of tea. It was an effective method to use organic fertilizer for acidified soil remediation to ensure tea yield and quality. In this study, different fertilizers were used to treat the acidified tea plantation soils for 4 consecutive years to analyze the remediation effect of different fertilizers on acidified soil and their effects on tea yield and quality. The results showed that during the period of 2017-2021, the soil pH value of tea plantation (S1) with long-term use of chemical fertilizer decreased continuously, from 3.07 to 2.82. In the tea plantation (S2), the soil pH value was stable between 4.26 and 4.65 in the combination of organic fertilizer and chemical fertilizer for a long time. The tea plantation (S3) with long-term use of organic fertilizer has a stable soil pH value between 5.13 and 5.33, which is most suitable for the growth of tea trees. The analysis results of tea yield and quality indicators (tea polyphenols, theanine, amino acids, caffeine, catechin components) showed that after long-term use of chemical fertilizer in S1 tea plantation, soil pH value decreased continuously, soil acidification intensified, tea tree growth was hindered, and tea yield and quality decreased continuously. S2 tea plantation used some organic fertilizer in combination with chemical fertilizer for a long time, the soil pH value gradually improved, soil acidification weakened, and tea yield and quality improved steadily. After long-term use of organic fertilizer in S3 tea plantation, soil acidification was significantly improved, which was conducive to the normal growth of tea trees and the yield and quality of tea reached the maximum. The results of interaction analysis showed that the long-term use of chemical fertilizer had a negative effect on the growth of tea trees, and the combination of organic fertilizer and chemical fertilizer improved the growth of tea trees to some extent, but the effect was poor, while the long-term use of organic fertilizer was the most beneficial to the growth of tea trees and most conducive to ensuring the yield and quality of tea. This study provides important practical significance for the remediation and fertilizer regulation of acidified tea plantation soils. In the process of field experiment, climate is a variable factor, and attention should be paid to the effect of climate change on fertilization efficiency in subsequent experiment.

## 1 Introduction

Tieguanyin (*Camellia sinensis*) tea tree is a perennial evergreen plant. Its origin is Anxi County, Fujian Province, China (24°50° – 25°26°N, 117°36° – 118°17°E). Tea trees are acid-loving plants. The soil pH value between 4.5 and 5.5 is suitable for tea tree planting, and the soil pH value >5.5 or <4.5 is not suitable for tea tree planting (Mohammad et al., 2014; Mehra and Baker, 2017). As a cash crop, tea trees are mainly harvested as shoots and young leaves, and therefore have a high demand for fertilizers, during growth (Liu et al., 2018). It had been reported that the annual use of chemical fertilizers for tea trees exceeds about two times the recommended amount of applied to tea plantations, and the heavy used of chemical fertilizers did not effectively ensure the yield and quality of tea, but rather exacerbates the degree of soil acidification (Akiyama et al., 2006; Ni et al., 2019).

The yield was the basis of tea planting, and the quality was the guarantee of economic benefits. The improvement of tea quality was of great significance to the improvement of tea economic benefits (Hazra et al., 2018; Bhargava et al., 2022). The heavy use of chemical fertilizers has caused soil acidification in tea plantations, which has led to a decline in the yield and quality of tea trees. For example, Lin et al. (2022) analyzed soil pH and its effect on tea yield and quality in 145 tea plantations in Nanjing County, Fujian Province. It was found that 82.1% of the tea plantations had soil pH < 4.5, and soil pH was significantly positively correlated with tea yield and quality, and soil acidification led to a significant decline in tea yield and quality. Wang et al. (2018) found that 37.67% of tea plantation soils in Anxi County, Fujian Province, China were acidified (pH <4.5). The yield and quality of tea trees tended to decrease after soil acidification (Wang et al., 2022). Yang et al. (Yang, 2021) investigated the soil of 5285 tea plantations in Anxi County and found that 68.44% of the tea plantations had soil pH<4.5. Secondly, it was found that the tea plantations with soil pH<4.5 mainly used chemical fertilizer, while the tea plantations used organic fertilizer had a higher soil pH value. It was considered that organic fertilizer played an important role in reducing soil acidification.

Organic fertilizer could effectively regulate soil acidification, improve the survival environment of soil microorganisms, increase soil microbial and soil enzyme activities, which in turn promote nutrient uptake and utilization of crops and guarantee crop yield and quality (Chepkorir et al., 2018; Chernov and Semenov, 2021; Prajna et al., 2022). Therefore, the use of organic fertilizer instead of chemical fertilizer was an effective way to improve soil acidification and an important path to achieve organic ecological agriculture. In recent years, numerous scholars have also carried out a large number of studies on soil acidification remediation by organic fertilizer, for example, rapeseed cake organic fertilizer (Xie et al., 2021), pig manure organic fertilizer (Ye et al., 2019; Cai et al., 2021), sheep manure organic fertilizer (Li et al., 2018; Le et al., 2022), cow manure organic fertilizer (Yin et al., 2022), commercial organic fertilizer (Lin et al., 2019; Wang et al., 2019) etc., to improve acidified soil. These studies confirmed that organic fertilizer could improve soil acidification.

In recent years, the adoption of organic fertilizer instead of chemical fertilizers has been vigorously promoted in Chinese tea plantations to ensure that the soil ecology of tea plantations could be sustained and healthy, and the choice of organic fertilizer was mainly sheep manure after fermentation. Sheep manure after fermentation is a kind of green ecological organic fertilizer. However, there were few reports on whether sheep manure fertilizer has obvious improvement effects on the tea plantation with severely acidified soil, and whether it has certain improvements on tea yield and quality, especially after long-term use. In this study, tea plantations with severely acidified soil were selected and treated with different fertilizers to analyze the changes in soil pH and its effects on tea yield and quality after using different fertilizers for four consecutive years (2017–2021), with a view to providing practical guidance for the improvement of acidified tea plantation soils and the regulation of tea plantation fertilization.

## 2 Material and methods

### 2.1 Field trial

**Figure 1:**
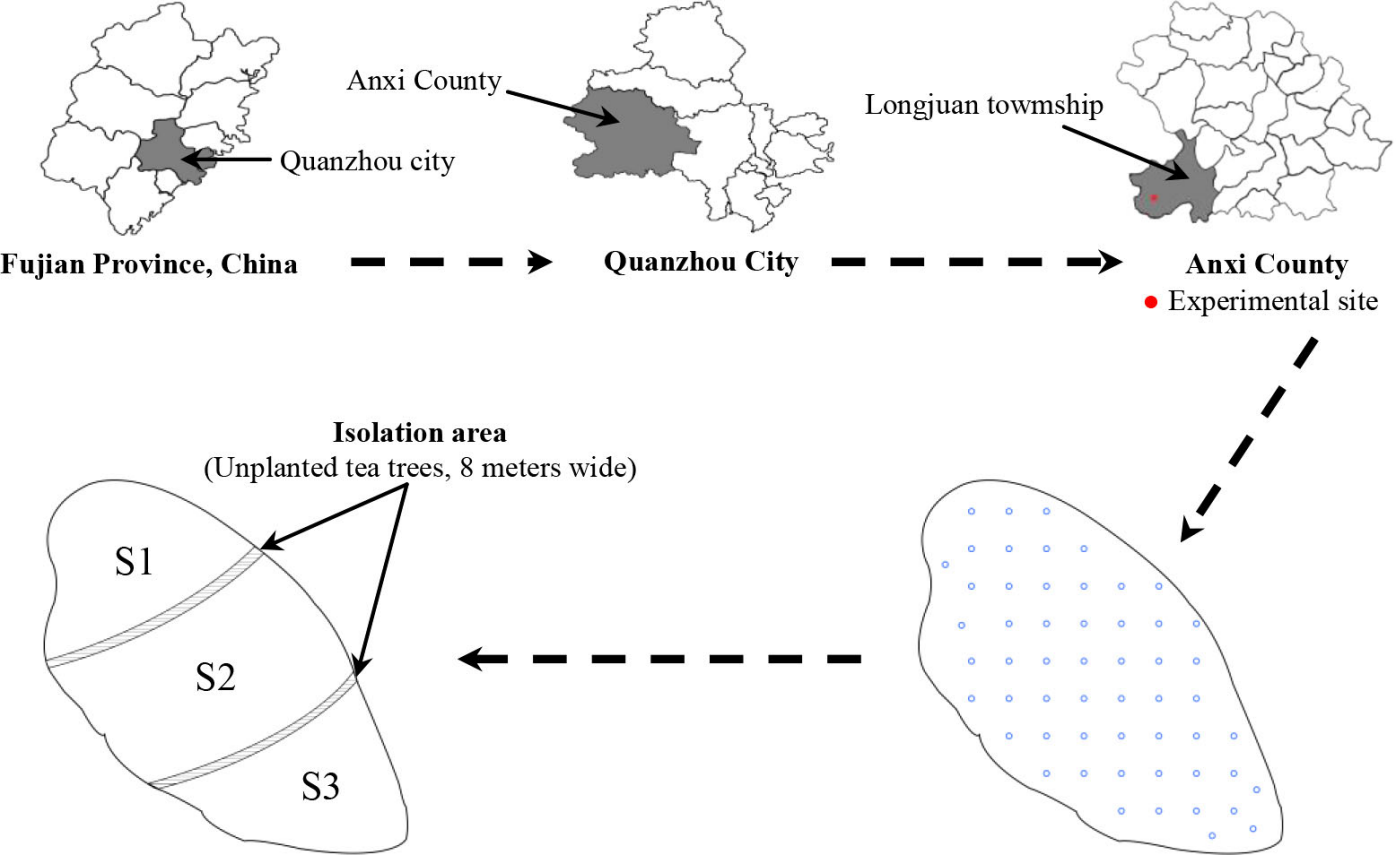
Schematic diagram of experimental site and tea plantation partition.

Anxi County, Quanzhou City, Fujian Province is the origin of Tieguanyin tea tree in China. The county area is 117°36'-118°17'E, 24°50'-25°26'N, with mean altitude of 600 m, annual rainfall of 1,800 mm, annual relative humidity of 80%, and average annual temperature of 18°C. Soil pH was suitable for tea planting when it was between 4.5 and 5.5, and when the pH was below 4.0, it was severe soil acidification (Mohammad et al., 2014; Mehra and Baker, 2017). Based on our previous study (Wang et al., 2018; Wang et al., 2022), Tieguanyin tea plantations located in Longjuan town (Longitude 117°93’ east and latitude 24°97’ north), Anxi County, Quanzhou City, Fujian Province, China was selected as experimental site (**Figure 1**).

The total area of the experimental tea plantation was 4.03ha, the tea trees were 3 years old, and the average pH value of the soil in the tea plantation was 3.29 (**Table S1**, **Figure S1**). The other physical and chemical indexes were 7.32 g/kg of organic matter, 2.63 g/kg of total nitrogen, 1.35 g/kg of total phosphorus, 1.76 g/kg of total potassium, 27.64 mg/kg of available nitrogen, 77.45 mg/kg of available phosphorus, 305.26 mg/kg of available potassium. The experimental tea plantation was divided into three areas, S1, S2 and S3, where S1 tea plantation covered 1.22 ha, S2 area 1.53 ha, and S3 area 1.28 ha (**Figure 1**). Fertilizer treatments were applied to tea plantations in different areas using different fertilizers for four consecutive years from November 2017 to November 2021 to analyze the effects of fertilizers on soil pH, tea yield and its quality index content in tea plantation.

### 2.2 Fertilization treatment method

The fermented sheep manure was used as organic fertilizer (1.64% total nitrogen, 0.91% total phosphorus, 0.89% total potassium) and the combined fertilizer was used as chemical fertilizer (nitrogen: urea; phosphate fertilizer: calcium superphosphate; potassium fertilizer: potassium sulfate). The nutrient, heavy metal and microbial indexes of fermented sheep manure are in line with the agricultural industry standard of the People’s Republic of China – “NY/T 525-2021 organic fertilizer” (Ministry of Agriculture of the People's Republic of China, 2021). The experiment was set up in three different areas S1, S2 and S3, where S1 was treated with 100% chemical fertilizer, S2 was treated with 50% organic fertilizer and 50% chemical fertilizer, S3 was treated with 100% organic fertilizer. The design of different fertilization treatments was based on the fertilization methods used by many tea farmers in the current tea plantation (Wang et al., 2020). The annual fertilizer dosage of the tea plantation was based on the application of 280 kg/ha of nitrogen, 150 kg/ha of phosphorus and 150 kg/ha of potassium and the different fertilizers were used according to the current pattern of fertilizer use in the tea plantation (Wu et al., 2015; Wang et al., 2018). Fertilizer treatments were applied to tea plantations in different areas for four consecutive years from November 2017 to November 2021 as follows (**Figure S2**, **Table S2**):

In S1 tea plantation, 100% chemical fertilizer was applied by evenly spreading in the planting process. The fertilizer was applied in November each year, with 45% of the annual amount fertilizer dosage; in March, with 30% of the annual fertilizer dosage; and in August,with 25% of the annual fertilizer dosage.

In S2 tea plantation, 50% organic fertilizer + 50% chemical fertilizer was used for fertilization treatment during the planting process. The fertilization time was: base fertilizer was applied in November every year, and all organic fertilizer was applied as base fertilizer. The fertilization method was ditching and burying on both sides of the tea planting field, and the amount of fertilizer accounts for 50% of the annual fertilizer amount. In March, accelerating budding fertilizer (chemical fertilizer) was applied evenly in the tea planting field, accounting for 25% of the annual fertilizer consumption. Topdressing (chemical fertilizer) was applied in August, and the fertilization method was evenly spread in the tea planting area, accounting for 25% of the annual fertilizer consumption.

In S3 tea plantation, 100% organic fertilizer was used for fertilization treatment. The method of fertilization was ditching and burying on both sides of tea planting sites. Fertilization time was November each year as the base fertilizer all applied.

### 2.3 Sample collection

The picking of Tieguanyin tea trees is divided into two periods each year. May is the spring tea picking period and October is the autumn tea picking period. Therefore, in this study, after different fertilization treatments, the S1, S2, and S3 areas were measured in May and October of each year (May 2018, October 2018, May 2019, October 2019, May 2020, October 2020, May 2021, and October 2021) for 4 consecutive years from November 2017 to November 2021, respectively. At the same time, fresh tea leaves (Functional leaves: on the branch of tea tree, the second leaf from the top bud down is called the functional leaf) and root circle soil of tea trees were collected from tea plantations of different areas. The soil in the root circle of tea tree was collected by removing the senescent leaves from the surface of the soil and digging the soil layer by layer until the root of tea tree (the depth is between 30 and 45 cm). The soil around the root of tea tree was collected for pH measurement. Among them, the collected leaves of tea tree were used for the determination of contents of tea polyphenols, theanine, amino acids, caffeine and catechin components, and the root circle soil of tea tree was used for the determination of soil pH.

### 2.4 Determination of soil pH

Soil pH was measured using a pH meter (PB-10, Sartorius) with 1:2.5 of the ratio of soil and water, and 5 replicates were used for each sample.

### 2.5 Determination of tea yield

Tea yield was determined with reference to the method of Wang et al. (2020). Specifically, the measurement time was in May (spring tea) and October (autumn tea) each year, the picking standard was 3-4 leaves in the middle and small open surface of the standing bud, the area of each sample was 10 m^2^ (1 row, length 10 m × width 1 m), and all 10 m^2^ were planted with tea trees, and three replicate samples were measured for each tea plantation of fertilization treatment. Based on the measured tea yield, the tea yield per hectare of tea plantation was converted.

### 2.6 Determination of tea quality index

The concentration of tea polyphenols, theanine, amino acids, caffeine and catechin components were determined in fresh leaves (functional leaves, second leaves) collected from tea trees, with 5 replicates for each sample. The extraction and detection methods of tea polyphenols and catechins were based on the National Standards of the People’s Republic of China (All China Federation of Supply and Marketing Cooperatives, 2019). The determination of theanine was based on the National standard of the People’s Republic of China using high performance liquid chromatography (General Administration of Quality Supervision, Inspection and Quarantine of the People's Republic of China, 2018). The determination of amino acids was based on the National standard of the People’s Republic of China using spectrophotometry and reaction with ninhydrin (General Administration of Quality Supervision, Inspection and Quarantine of the People's Republic of China, 2014b). The determination of caffeine was based on the National Standards of the People’s Republic of China (General Administration of Quality Supervision, Inspection and Quarantine of the People's Republic of China, 2014a).

### 2.7 Analysis

Excel 2017 software was used to calculate the average value and variance of data and to make bar charts, Heml 1.0 software was used to make heat maps, Origin 2018 software was used to make trend charts, Rstudio 3.3 software was used to make principal component maps, and Gephi 0.9.2 software was used to make relational network charts. Cytoscape_v3.9.1 software was used to make the correlation analysis chart.

## 3 Results and discussion

### 3.1 Effect of fertilization treatment on soil pH

**Figure 2:**
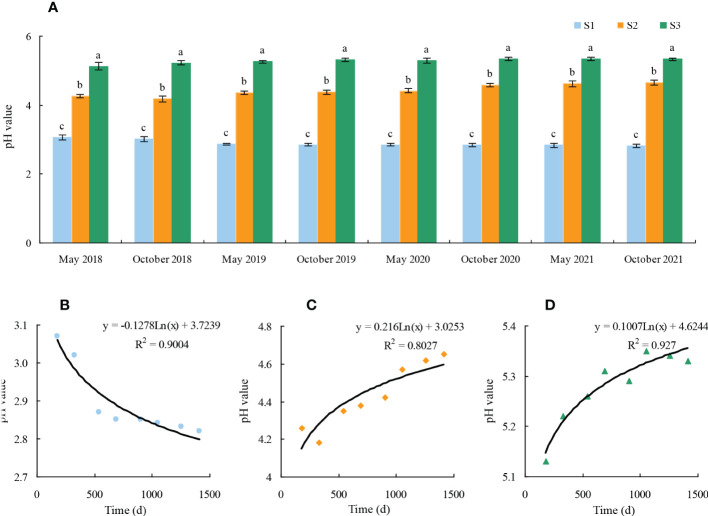
Effect of different fertilization treatments on soil pH in tea plantation S1: 100% Chemical fertilizer; S2:50% Organic fertilizer +50% chemical fertilizer; S3: 100% Organic fertilizer; **(A)** Effect of 4 consecutive years of fertilization on soil pH in tea plantation; **(B)** Trend analysis of soil pH after 4 years of continuous treatment in S1 tea plantation; **(C)** Trend analysis of soil pH after 4 years of continuous treatment in S2 tea plantation; **(D)** Trend analysis of soil acidity after 4 years of continuous treatment in S3 tea plantation; Different lowercase letters indicate the significant difference at *P* < 0.05 levels among different treatments.

Tea trees are acid-loving plants. The soil pH value between 4.5 and 5.5 is suitable for tea tree planting, and the soil pH value between 5.0 and 5.5 is the most suitable for tea tree planting. When the soil pH value was between 4.0 and 4.5, it was considered as mild soil acidification, and when the pH value was below 4.0, it was considered as severe soil acidification (Mohammad et al., 2014; Mehra and Baker, 2017; Lin et al., 2022). Soil acidification destroyed the root system of tea trees and seriously affected their growth, which in turn led to lower tea yield and quality, and lower economic benefits (Wen et al., 2021; Yang et al., 2021; Le et al., 2022; Tang et al., 2022). In this study, the effect of different fertilization treatments on soil pH in tea plantations for four consecutive years was analyzed, and the results showed (**Figure 2**) that fertilization significantly changed soil pH in tea plantations, and the difference in soil pH between different fertilization treatments reached a significant level, with the highest soil pH in tea plantations using all organic fertilizers and the lowest in those using all chemical fertilizers (**Figure 2A**). Secondly, it was found that S1 tea plantation with long-term use of chemical fertilizer (2017–2021) had a continuous decrease in soil pH from 3.07 to 2.82, with increasing soil acidification and unsuitable for tea tree growth (**Figure 2B**). The S2 tea plantation with long-term use of some organic fertilizers in combination with chemical fertilizers (2017–2021) had a stable soil pH of 4.26 to 4.65, with some improvement in soil pH and basically suitable for tea tree growth(**Figure 2C**). And S3 tea plantation with long-term use of organic fertilizer (2017–2021) had a stable soil pH between 5.13 and 5.33, which was most suitable for tea tree growth (**Figure 2D**). Yan et al. (2020) analyzed the current situation of tea plantations in China and concluded that the soil is generally acidified, while the degree of soil acidification in tea plantations using organic fertilizers is relatively low and organic fertilizers are beneficial to soil improvement. Yang et al. (2018) studied the effect of long-term use of chemical fertilizers on soil acidification in tea plantations and found that long-term use of nitrogen fertilizers led to increased soil acidification, accelerated nitrogen loss from the soil, and growth of tea trees was hindered. Xie et al. (2021) compared the effects of compound fertilizer and rape cake organic fertilizer on soil acidification in tea plantations and found that rape cake organic fertilizer was conducive to reduction of acidification in a tea plantation soil, and was conducive to the growth and quality improvement of tea trees. It can be seen that appropriate use of organic fertilizer in tea plantation was conducive to improving soil acidification, and the long-term use of organic fertilizer was the most effective for reduction of acidification in a tea plantation soil and guaranteeing the growth of tea trees at the optimal soil pH value.

### 3.2 Effect of fertilization treatment on tea yield

**Figure 3:**
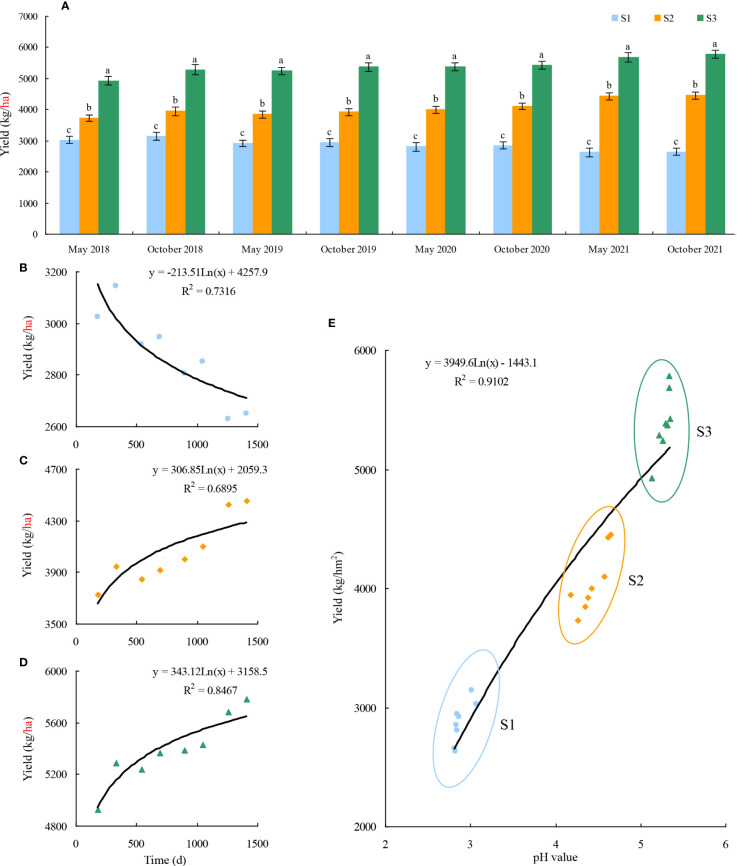
Effects of different fertilization treatments and soil pH on tea yield. S1: 100% Chemical fertilizer; S2:50% Organic fertilizer +50% chemical fertilizer; S3: 100% Organic fertilizer; **(A)** Effect of fertilization treatment on tea yield in four consecutive years; **(B)** Trend analysis of the effect of continuous treatment for 4 years on tea yield in S1 tea plantation; **(C)** Trend analysis of the effect of continuous treatment for 4 years on tea yield in S2 tea plantation; **(D)** Trend analysis of the effect of continuous treatment for 4 years on tea yield in S3 tea plantation; **(E)**: Trend analysis of influence of soil pH on the tea yield. Different lowercase letters indicate the significant difference at *P* < 0.05 levels among different treatments.

Tieguanyin is one of the top ten famous teas in China, and Anxi County is the origin of Tieguanyin. In 2021, Anxi County tea plantation covered an area of 40,000 ha, produced 62,000 t, and the total output value of tea was 28 billion RMB. Anxi Tieguanyin ranked first in the national tea category with a brand value of 142.846 billion RMB for 6 consecutive years and first in the national key tea-producing counties for 10 consecutive years. In May 2021, Anxi Tieguanyin tea culture system was recognized by the Food and Agriculture Organization of the United Nations as a globally important agricultural cultural heritage (Minnan Network. Anxi, 2022). Therefore, the security of tea production is of great significance for the development of tea industry in Anxi County. In this study, the effects of long-term different fertilizer treatment and soil pH on tea yield were analyzed, and the results showed (**Figures 3A–E**) that the fertilization can significantly affect tea yield, and the differences in tea yield between different fertilization treatments reached significant level, with the highest tea yield in tea plantations using organic fertilizers and the lowest in those using chemical fertilizers (**Figures 3A**). Secondly, it was found that S1 tea plantation with long-term use of chemical fertilizer (2017–2021) had a continuous decrease in tea productivity, i.e., from 3145 kg/ha to 2630 kg/ha (**Figure 3B**). S2 tea plantation with long-term use of organic fertilizer in combination with chemical fertilizer (2017–2021) had a significant increase in tea productivity, i.e., from 3729 kg/ha to 4452 kg/ha (**Figure 3C**). And S3 tea plantation with long-term use of organic fertilizer (2017–2021) had the highest tea yield and the most obvious improvement, which showed an increase from 4928 kg/ha to 5782 kg/ha (**Figure 3D**). Further fitting analysis of soil pH value and tea yield with different fertilizers use found (**Figure 3E**) that soil pH value was significantly and positively correlated with tea yield. It can be seen that after long-term use of chemical fertilizer in S1 tea plantation, soil pH value continuously decreased, soil acidification intensified, and tea yield decreased; after long-term use of organic fertilizers with chemical fertilizers in S2 tea plantation, soil pH value gradually improved, soil acidification weakened, and tea yield steadily increased; after long-term use of organic fertilizer in S3 tea plantation, soil acidification significantly improved and tea yield reached the maximum. In conclusion, when applying fertilizer in tea plantation, the appropriate use of organic fertilizer is not only beneficial to improve soil acidification, but also to increase tea yield.

### 3.3 Effect of fertilization treatment on tea quality

**Figure 4:**
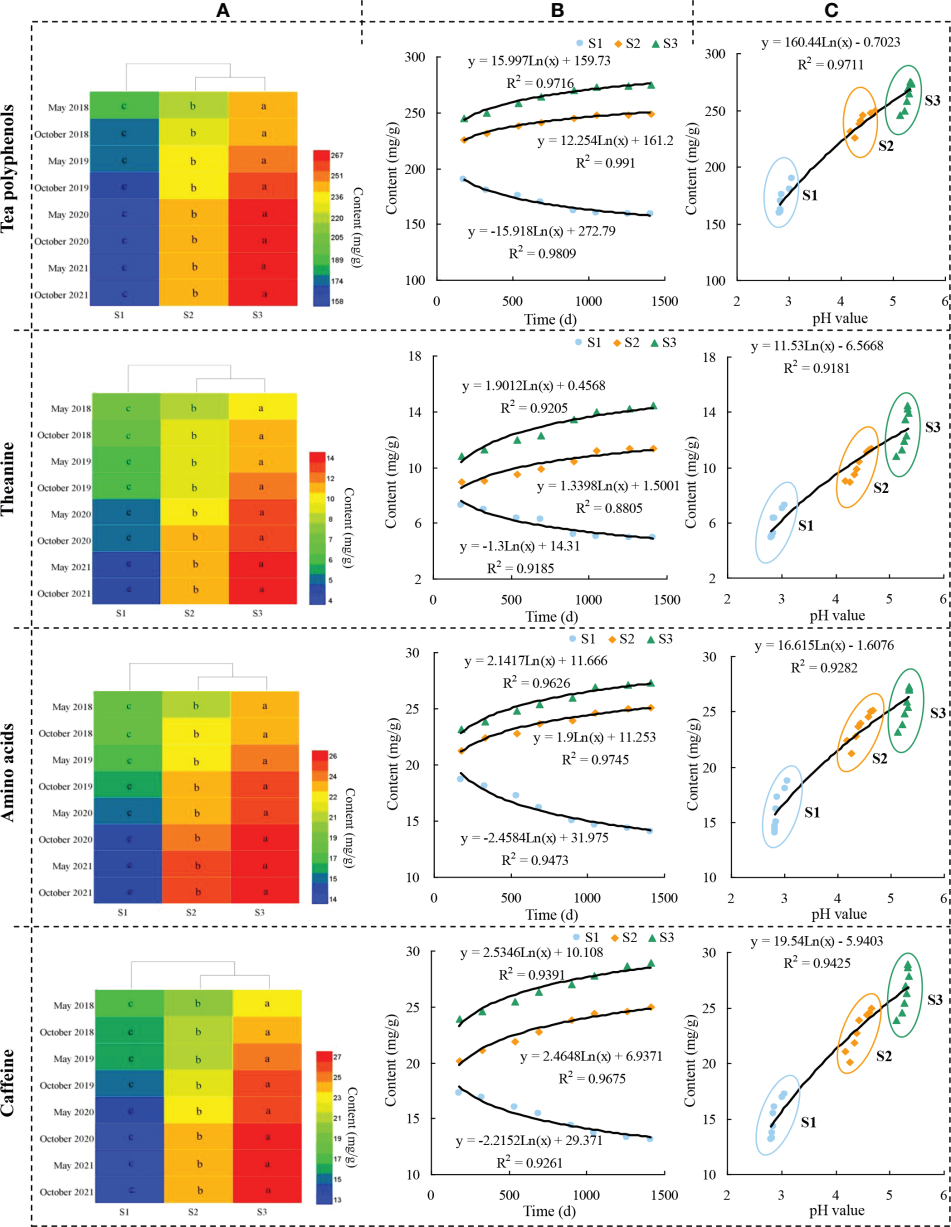
Effects of different fertilization treatments and soil pH on the content of tea leaf quality indexes. Note: S1: 100% Chemical fertilizer; S2: 50% Organic fertilizer +50% chemical fertilizer; S3: 100% Organic fertilizer; **(A)**: Effect of 4 consecutive years of fertilization treatment on the quality tea leaves; **(B)** Trend analysis of influence of fertilization treatment on the quality tea leaves after 4 consecutive years; **(C)** Trend analysis of influence of soil pH on the quality tea leaves. Different lowercase letters indicate the significant difference at P < 0.05 levels among different treatments.

**Figure 5:**
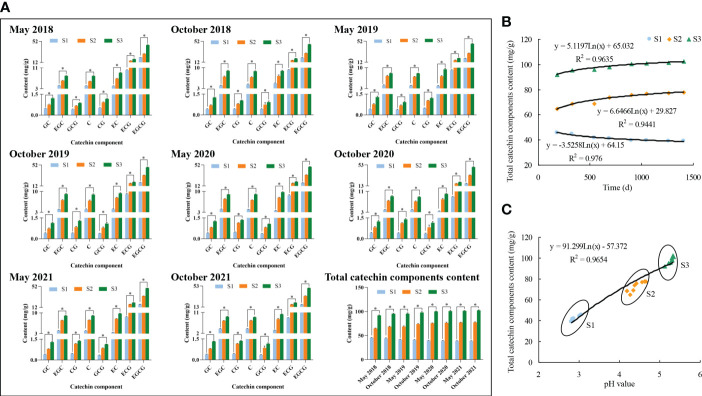
Effects of different fertilization treatments and soil pH on the content of catechin components in tea leaves. Note: S1: 100% Chemical fertilizer; S2:50% Organic fertilizer +50% chemical fertilizer; S3: 100% Organic fertilizer; GC, Gallocatechin; EGC, Epigallocatechin; CG, Catechin gallat; C, Catechin+C; GCG, Gallocatechin gallate; EC, Epicatechin; ECG, Epicatechin gallat; EGCG, Epigallocatechin gallate; **(A)** Changes of catechin content in tea leaves after continuous fertilization; **(B)** Trend analysis of the effect of 4 consecutive years of fertilization on the content of catechin components in tea leaves; **(C)** Trend analysis of the influence of soil pH on the content of catechin components in tea leaves; * indicate the significant difference at P < 0.05 levels among different treatments.

The yield was the basic of tea tree planting and the quality was the guarantee of economic benefits. The improvement of tea quality was of great significance for the improvement of tea economic benefits (Hazra et al., 2018; Bhargava et al., 2022). Theanine, caffeine and polyphenols were secondary metabolites of tea tree, and were important quality components that constituted the taste and aroma characteristics. Amino acids, as primary metabolites of the tea tree, played an important role in the formation of umami and sweet taste of tea soup (Miyauchi et al., 2014; Ye et al., 2016; Jia et al., 2018; Chen et al., 2021). Many scholars usually used tea polyphenols, theanine, amino acids and catechin components as evaluation indicators in evaluating the effect of biotic or abiotic stress on tea quality, and their high content means good quality and vice versa (Yan et al., 2020; Wen et al., 2021; Le et al., 2022; Tang et al., 2022). Accordingly, this study evaluated the effects of different fertilization treatments and soil acidification on the quality of tea leaves with the above mentioned indicators, and the results showed (**Figures 4**, **5**) that fertilization had a significant effect on the content of quality indexes (tea polyphenols, theanine, amino acids, caffeine, and catechin components) of tea leaves, and the differences in the content of quality indexes among different fertilization treatments reached a significant level, with the highest content of tea leaf quality indexes still found in tea plantations using organic fertilizers and the lowest in those using chemical fertilizers (**Figures 4A**, **5A**).

Further analysis of the effects of long-term different fertilization treatments on the content of quality indicators of tea leaves revealed (**Figures 4B**, **5B**) that the long-term use of chemical fertilizers in S1 tea plantation (2017–2021) showed a significant decrease in quality index content of tea leaves, as shown by a decrease in tea polyphenols content from 189.67 mg/g to 158.84 mg/g, theanine content decreased from 7.62 mg/g to 4.91 mg/g, amino acid content decreased from 18.65 mg/g to 14.03 mg/g, caffeine content decreased from 17.23 mg/g to 13.07 mg/g, and the total catechin content decreased from 45.54 mg/g to 38.96 mg/g. S2 tea plantation used organic fertilizer in combination with chemical fertilizer for a long time (2017–2021), and the content of quality indexes of tea leaves increased significantly, as shown by the increase of tea polyphenols content from 225.36 mg/g to 249.25 mg/g, theamine content increased from 8.93 mg/g to 11.38 mg/g, amino acid content increased from 21.25 mg/g to 25.12 mg/g, caffeine content increased from 20.14 mg/g to 24.97 mg/g, and the total catechin content increased from 64.96 mg/g to 77.64 mg/g. While S3 tea plantation used organic fertilizers for a long time (2017–2021) had the highest quality index content and the most obvious increase, as shown by the increase in tea polyphenols content from 245.37 mg/g to 274.95 mg/g, theamine content from 10.85 mg/g to 14.74 mg/g, amino acid content from 23.16 mg/g to 27.31 mg/g, caffeine content from 23.98 mg/g to 28.93 mg/g, and the total catechin content from 91.89 mg/g to 102.51 mg/g.

Further fitting analysis between soil pH value after different fertilizers and tea leaves quality index content found (**Figures 4C**, **5C**) that soil pH value was significantly and positively correlated with tea leaves quality index content, which increased significantly with the increase of soil pH value. The results showed that after long-term use of chemical fertilizer in S1 tea plantation, soil pH value continuously decreased, soil acidification intensified, tea tree growth was hindered, and tea quality decreased; S2 tea plantation used some organic fertilizer in combination with chemical fertilizer for a long time, the soil pH value gradually improved, soil acidification weakened, and tea quality improved steadily; after long-term use of organic fertilizer in S3 tea plantation, soil acidification was significantly improved, which was conducive to the normal growth of tea trees and the quality of tea leaves reached the maximum. It can be seen that the appropriate use of organic fertilizer in tea plantation is not only conducive to improving soil acidification, but also to improving tea quality.

### 3.4 Interaction analysis of different indexes under fertilization treatment

**Figure 6:**
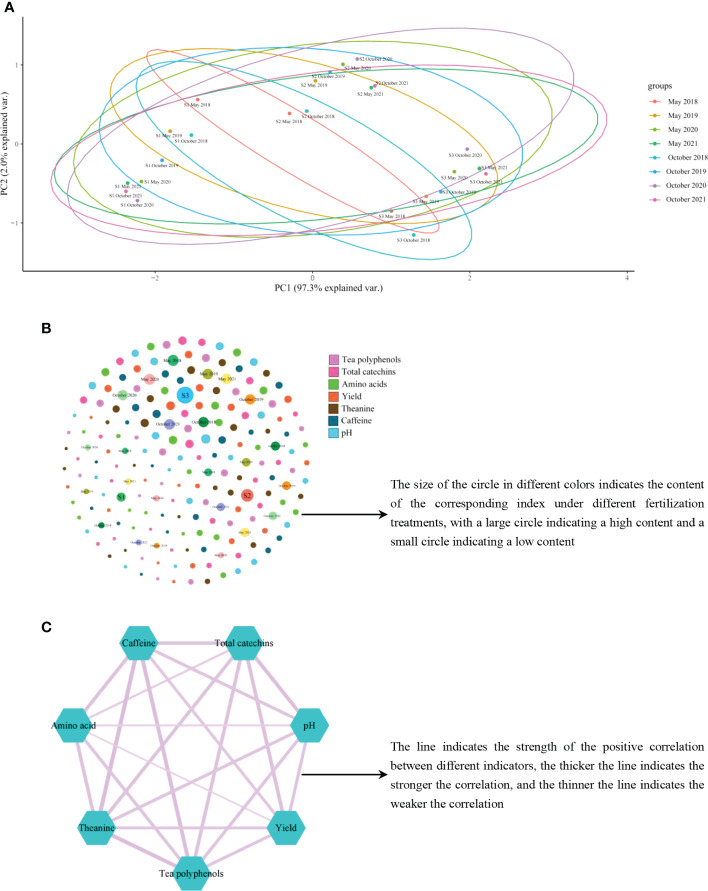
Interactions among soil pH, tea yield and quality indexes after different fertilization treatments. S1: 100% Chemical fertilizer; S2:50% Organic fertilizer +50% chemical fertilizer; S3: 100% Organic fertilizer; **(A)** Principal component analysis of samples from different years after different fertilization treatments; **(B)** Relationship network analysis of all measured indexes in different year samples after different fertilization treatments; **(C)** Correlation analysis of all measured indexes in different year samples after different fertilization treatments.

In this study, PCA, relationship network analysis and correlation analysis were used to explore the interactions among soil pH, tea yield and tea quality after different fertilization treatments for 4 consecutive years. The results of PCA showed (**Figure 6A**) that after long-term application of different fertilization treatments (2017–2021), the gap between the measured indicators increased with time, which was reflected in the continuous increase of the area between different circles, specifically October 2021 > May 2021 > October 2020 > May 2020 > October 2019 > May 2019 > October 2018 > May 2018. Secondly, S1 tea plantation with long-term use of chemical fertilizers was located at the negative end of PC1, and its negative value increased with the extension of time. S2 tea plantation that used some organic fertilizers in combination with chemical fertilizers for a long time, was located at the junction of the positive and negative ends of PC1, and with the extension of time, it changed from negative to positive and keeped increasing. S3 tea plantation with long-term use of organic fertilizer was located at the positive end of PC1, and its positive value increased with the extension of time. The results (**Figure 6B**) showed that soil pH value, tea yield and quality index content were always the largest in S1 tea plantation, the second largest in S2 tea plantation, and the smallest in S3 tea plantation under different fertilization treatments. The results of correlation analysis showed (**Figure 6C**; **Table S3**) that after different fertilization treatments, the pH value of tea plantation soil was positively correlated with each index, that is, the value of each index increased with the increase of pH value, and decreased otherwise. It can be seen that the long-term use of chemical fertilizer has a negative effect on the growth of tea trees, and the use of organic fertilizer in combination with chemical fertilizer could improve the growth of tea trees to some extent, but the effect was poor, while the long-term use of organic fertilizer was most conducive to the growth of tea trees, and was most conducive to ensuring the yield and quality of tea trees.

## 4 Conclusion

**Figure 7:**
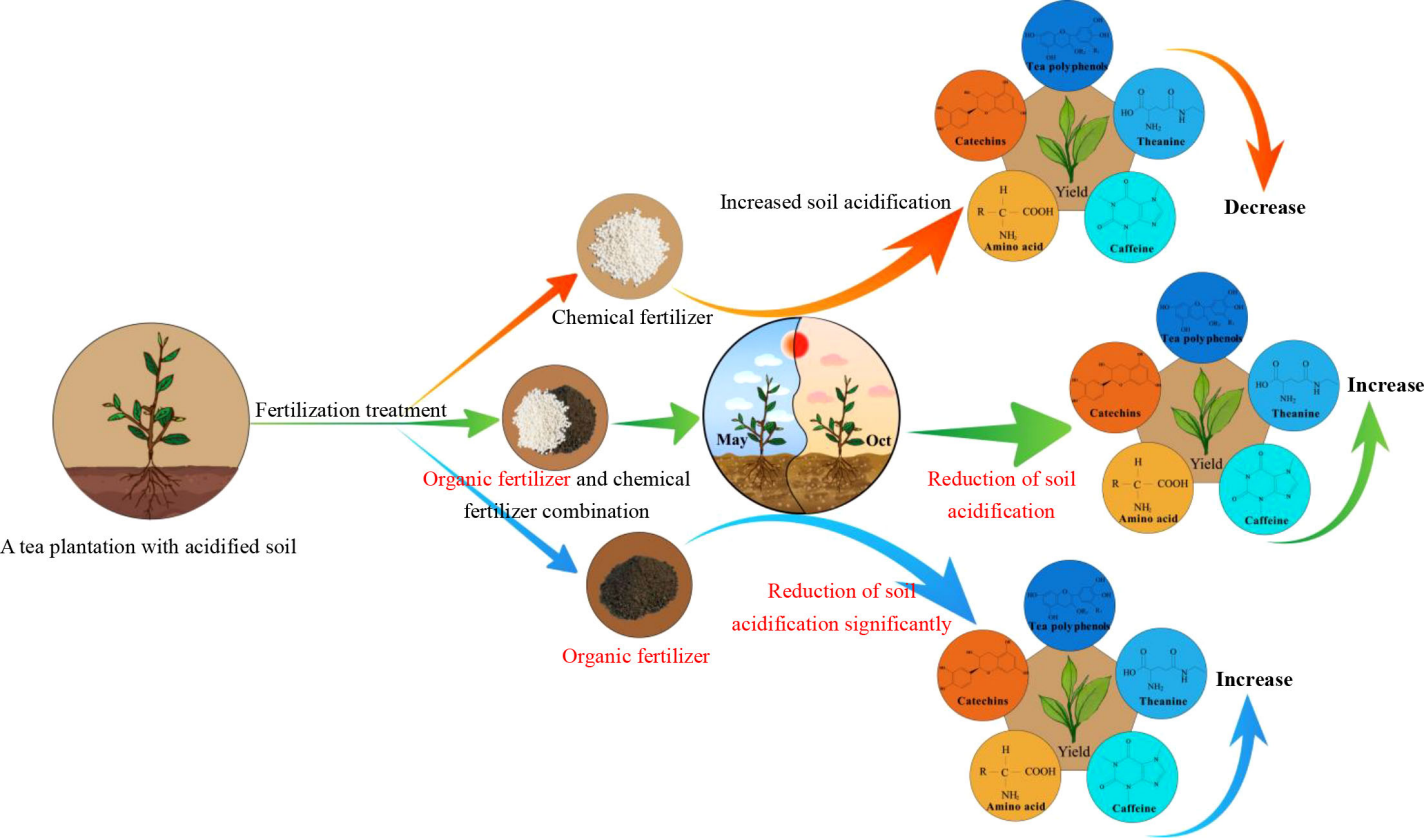
Effects of different fertilizer treatments on soil pH, tea yield (spring tea in May, autumn tea in October) and quality in tea plantation. Chemical fertilizer can aggravate soil pH and reduce tea yield and quality. Organic fertilizer combined with chemical fertilizer can alleviate soil acidification and improve the yield and quality of tea. Organic fertilizer treatment can effectively improve soil acidification in tea plantations and significantly improve tea yield and quality.

In this study, the effect of reduction of soil acidification in tea plantations and its effect on tea yield and quality were analyzed after using different fertilizers continuously (2017 to 2021) on the already acidified tea plantation soil. The results showed (**Figure 7**) that soil acidification in tea plantations could seriously affect the yield and quality of tea. Secondly, the long-term use of chemical fertilizers (S1) would aggravate soil acidification in tea plantations, which in turn would lead to a continuous decrease in tea yield and quality. The combination of organic fertilizer and chemical fertilizer (S2) could alleviate soil acidification and improve tea yield and quality to a certain extent, but the effect was poor. Of course, the mixing ratio of organic fertilizer and chemical fertilizer used in this study was 1:1, which was the fertilization method adopted by many tea farmers at present. Different responses are expected when using different fertilizer ratios. In contrast, long-term use of organic fertilizer (S3) could significantly improve soil acidification, tea yield and quality. This study provided an important practical basis for reduction of acidification in a tea plantation soil, and fertilizer management in tea plantations. However, climate is an objective and variable factor in the field experiment, and attention should also be paid to the effects of climate change on fertilizer application in subsequent studies.

## Data availability statement

The original contributions presented in the study are included in the article/**Supplementary Material**. Further inquiries can be directed to the corresponding authors.

## Author contributions

JY and YuhW: Conceptualization, Visualization, Methodology, Writing – original draft, Formal analysis, Writing – review & editing, Funding acquisition. YucW and LH; Formal analysis, Writing – review & editing. XJ and YucW: Methodology, Investigation, Writing – original draft. JY ang SL: Formal analysis, Writing – review & editing. LH and JK: Methodology, Investigation. HW and ZW: Conceptualization, Visualization, Methodology, Writing – original draft, Formal analysis, Writing – review & editing, Funding acquisition. All authors contributed to the article and approved the submitted version.
