# Supplementary material for: Improvement of soil acidification in tea plantations by long-term use of organic fertilizers and its effect on tea yield and quality
Source: Front Plant Sci. 2022 Dec 23;13:1055900. doi: 10.3389/fpls.2022.1055900 (PMC9822707; doi:10.3389/fpls.2022.1055900)
Supplement: Supplementary file 1 [file DataSheet_1.pdf]

Supplementary Materials

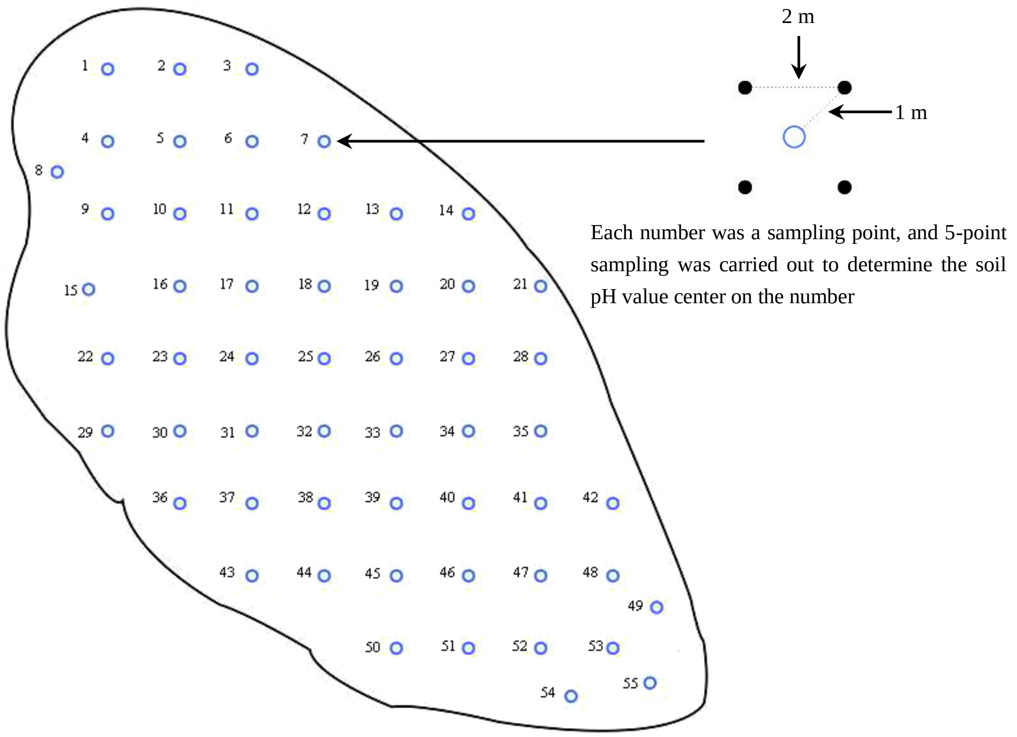

Fig. S1 Sampling distribution map of soil pH value determination samples in tea plantation in experimental field

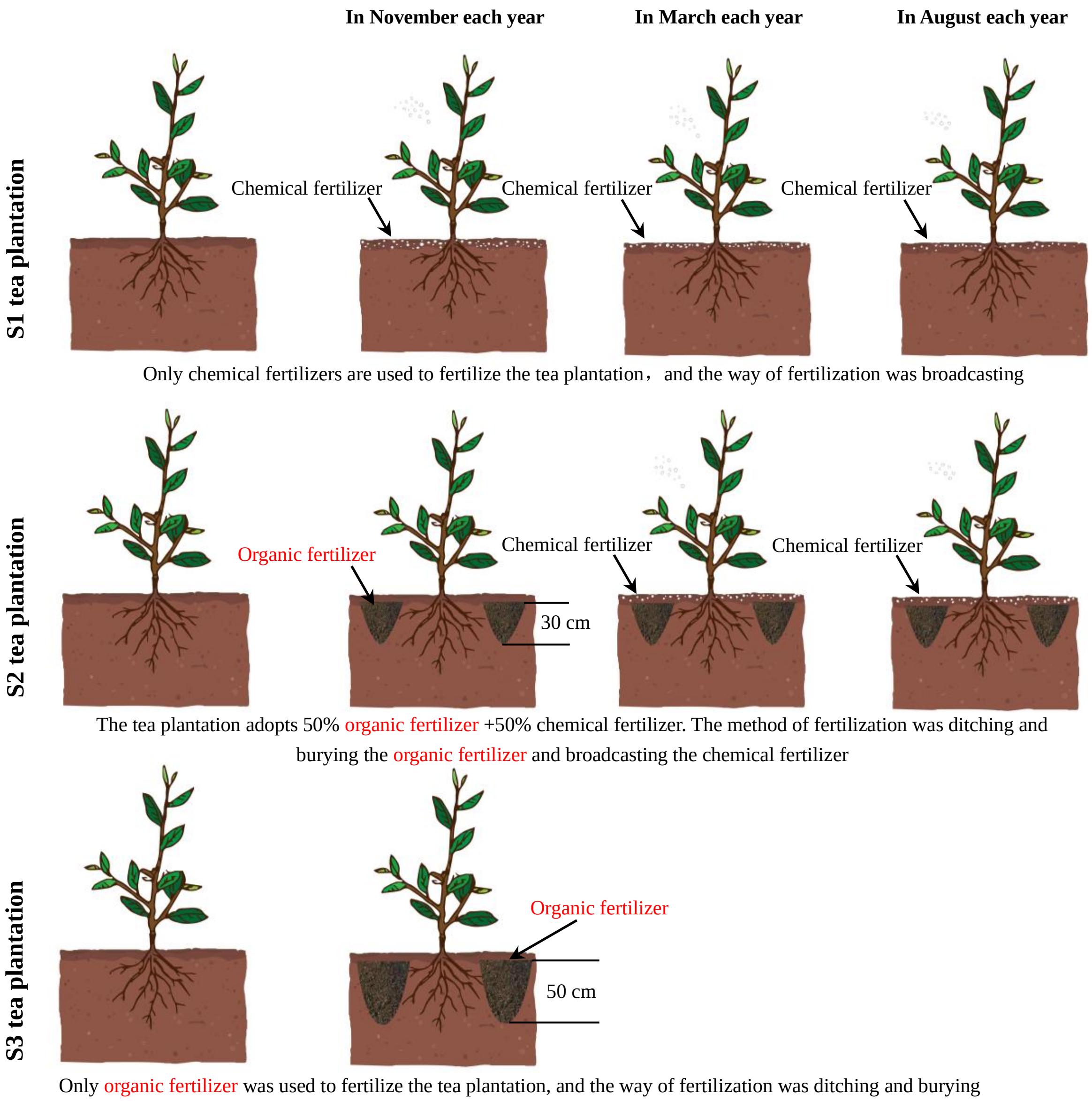

Fig. S2 Schematic diagram of tea tree fertilization

Note: S1: 100% Chemical fertilizer; S2:50% Organic fertilizer +50% chemical fertilizer; S3: 100% Organic fertilizer;

Table. S1 Soil pH value of tea plantation in the experiment site

No.	pH value	No.	pH value	No.	pH value	No.	pH value	No.	pH value
**1**	3.28±0.05	**12**	3.24±0.01	**23**	3.18±0.02	**34**	3.31±0.03	**45**	3.37±0.02
**2**	3.24±0.04	**13**	3.27±0.02	**24**	3.35±0.02	**35**	3.34±0.01	**46**	3.38±0.03
**3**	3.39±0.03	**14**	3.29±0.04	**25**	3.42±0.01	**36**	3.26±0.04	**47**	3.23±0.02
**4**	3.18±0.02	**15**	3.38±0.03	**26**	3.36±0.03	**37**	3.28±0.03	**48**	3.19±0.03
**5**	3.38±0.03	**16**	3.42±0.02	**27**	3.34±0.02	**38**	3.26±0.04	**49**	3.36±0.04
**6**	3.25±0.04	**17**	3.26±0.05	**28**	3.35±0.04	**39**	3.21±0.06	**50**	3.37±0.03
**7**	3.36±0.02	**18**	3.28±0.04	**29**	3.25±0.03	**40**	3.24±0.02	**51**	3.22±0.05
**8**	3.27±0.03	**19**	3.25±0.03	**30**	3.23±0.05	**41**	3.23±0.04	**52**	3.21±0.02
**9**	3.29±0.04	**20**	3.24±0.05	**31**	3.28±0.02	**42**	3.22±0.05	**53**	3.24±0.03
**10**	3.26±0.05	**21**	3.34±0.02	**32**	3.35±0.03	**43**	3.25±0.03	**54**	3.26±0.04
**11**	3.28±0.04	**22**	3.41±0.03	**33**	3.37±0.02	**44**	3.27±0.04	**55**	3.21±0.04
**The range of pH value**						3.18 ~ 3.42			
**The average pH value**						3.29			
**Standard deviation of pH value**						0.07			
**Coefficient of variation in pH value**						2.02			

Table S2 Fertilization time and fertilizer use in different experimental site as a percentage of the total annual fertilization

**Experimental site**	**First year**			**Second year**			**Third year**			**Fourth year**		
	November 2017	March 2018	August 2018	November 2018	March 2019	August 2019	November 2019	March 2020	August 2020	November 2021	March 2021	August 2021
S1	45%	30%	25%	45%	30%	25%	45%	30%	25%	45%	30%	25%
S2	50%	25%	25%	50%	25%	25%	50%	25%	25%	50%	25%	25%
S3	100%			100%			100%			100%		

Note: S1: 100% Chemical fertilizer; S2:50% Organic fertilizer +50% chemical fertilizer; S3: 100% Organic fertilizer;

Table S3 Correlation analysis between soil pH value and tea yield and quality

	pH	Yield	Tea polyphenols	Theanine	Amino acid	Caffeine
Yield	0.985**					
Tea polyphenols	0.986**	0.984**				
Theanine	0.985**	0.982**	0.997**			
Amino acid	0.963**	0.959**	0.986**	0.991**		
Caffeine	0.985**	0.982**	0.997**	1.000**	0.991**	
Total catechins	0.995**	0.993**	0.991**	0.990**	0.969**	0.990**
